# Supplementary material for: Diagnostic accuracy of the atherogenic index of plasma in metabolic syndrome: a diagnostic meta-analysis
Source: PeerJ. 2025 Oct 7;13:e20074. doi: 10.7717/peerj.20074 (PMC12513372; doi:10.7717/peerj.20074)
Supplement: Supplemental Information 2 [file peerj-13-20074-s002.docx]

1. The rationale for conducting the meta-analysis

Metabolic syndrome is a global health problem, with a global prevalence of 12.5% to 31.4% among adults, with the highest prevalence in the Eastern Mediterranean and the Americas (Dobrowolski et al. 2022). The prevalence increases with national income, and early screening and diagnosis are very important for preventing cardiovascular disease. It is important to understand the diagnosis of MetS (Saito et al. 2025), and a simple, non-invasive, and economical diagnostic tool is needed. The existing diagnosis method of MetS relies on the comprehensive evaluation of multiple indicators, such as waist circumference, blood pressure, fasting blood sugar, which is complicated to operate and time-consuming. Hence, it is necessary to find simple, fast, and accurate diagnostic indicators (Mameli et al. 2017).

The calculation method of AIP is simple and direct, requiring only two indicators, triglycerides and high-density lipoprotein cholesterol, which are components of routine blood lipid examinations (Jiang et al. 2025). This means that no additional examination items are required, and existing test data can be fully utilized, reducing the use of medical resources. At the same time, the calculation method of AIP is standardized and not affected by subjective factors of the measurer, which is conducive to the comparison and communication of results between different medical institutions (Jiang et al. 2025). This objectivity and repeatability are very important for clinical research and practice. In addition, since AIP uses routine blood lipid examination data, it does not require additional special examination equipment or reagents. Therefore, it will not increase the economic burden on patients. This high cost-effectiveness makes it particularly suitable for primary medical institutions and large-scale population screening (Kaneva et al. 2025). However, recent studies have reported large differences in the accuracy of AIP in diagnosing MetS. For example, the AUC for AIP in diagnosing MetS was 0.914 in one study (Vega-Cardenas et al. 2023)and 0.716 in another study (Zhang et al. 2021).

Therefore, it is necessary to systematically evaluate the accuracy of AIP in the diagnosis of MetS through a meta-analysis to provide reliable evidence for clinical practice.

2. The contribution that it makes to knowledge in light of previously published related reports, including other meta-analyses and systematic reviews

First, the calculation of AIP is based on serum lipid levels, but lipid metabolism is affected by many factors, such as diet, genetics, and lifestyle, which may lead to large inter-individual differences in AIP values (Yuan et al. 2024). Second, the results of studies on the correlation between AIP and metabolic syndrome are inconsistent, and there is a lack of unified diagnostic criteria (Babaahmadi-Rezaei et al. 2024). This study is the first systematic review and meta-analysis of the accuracy of AIP in the diagnosis of MetS. It includes studies based on multiple populations and regions to improve the representativeness of evidence and explores factors affecting diagnostic efficacy through subgroup analysis. The conclusions of this study can provide doctors with evidence-based medicine and help optimize MetS screening strategies. In addition, the research results can provide a reference for the formulation of relevant guidelines and public health policies.

**Reference**

Babaahmadi-Rezaei H, Raeisizadeh M, Zarezade V, Noemani K, Mashkournia A, and Ghaderi-Zefrehi H. 2024. Comparison of atherogenic indices for predicting the risk of metabolic syndrome in Southwest Iran: results from the Hoveyzeh Cohort Study (HCS). *Diabetology & Metabolic Syndrome* 16. 10.1186/s13098-024-01349-1

Dobrowolski P, Prejbisz A, Kuryłowicz A, Baska A, Burchardt P, Chlebus K, Dzida G, Jankowski P, Jaroszewicz J, Jaworski P, Kamiński K, Kapłon-Cieślicka A, Klocek M, Kukla M, Mamcarz A, Mastalerz-Migas A, Narkiewicz K, Ostrowska L, Śliż D, Tarnowski W, Wolf J, Wyleżoł M, Zdrojewski T, Banach M, Januszewicz A, and Bogdański P. 2022. Metabolic syndrome - a new definition and management guidelines: A joint position paper by the Polish Society of Hypertension, Polish Society for the Treatment of Obesity, Polish Lipid Association, Polish Association for Study of Liver, Polish Society of Family Medicine, Polish Society of Lifestyle Medicine, Division of Prevention and Epidemiology Polish Cardiac Society, "Club 30" Polish Cardiac Society, and Division of Metabolic and Bariatric Surgery Society of Polish Surgeons *Arch Med Sci* 18:1133-1156. 10.5114/aoms/152921

Jiang S, Liu S, Xiao G, Liu K, and Li J. 2025. Atherogenic index of plasma and the clinical outcome of patients with acute coronary syndrome: a meta-analysis. *Ann Med* 57:2442532. 10.1080/07853890.2024.2442532

Kaneva AM, Potolitsyna NN, and Bojko ER. 2025. The triglyceride-glucose index as an indicator of latent atherogenicity of the plasma lipid profile in healthy men with normolipidaemia. *Postgrad Med* 137:93-99. 10.1080/00325481.2024.2426970

Mameli C, Zuccotti GV, Carnovale C, Galli E, Nannini P, Cervia D, and Perrotta C. 2017. An update on the assessment and management of metabolic syndrome, a growing medical emergency in paediatric populations. *Pharmacol Res* 119:99-117. 10.1016/j.phrs.2017.01.017

Saito I, Yamagishi K, Kokubo Y, Yatsuya H, Muraki I, Iso H, Inoue M, Tsugane S, and Sawada N. 2025. Lifetime Risk of Incident Coronary Heart Disease, Stroke, and Cardiovascular Disease: The Japan Public Health Center-Based Prospective Study. *J Atheroscler Thromb* 32:48-57. 10.5551/jat.64934

Vega-Cardenas M, Teran-Garcia M, Manuel Vargas-Morales J, Padron-Salas A, and Aradillas-Garcia C. 2023. Visceral adiposity index is a better predictor to discriminate metabolic syndrome than other classical adiposity indices among young adults. *American Journal of Human Biology* 35. 10.1002/ajhb.23818

Yuan Y, Shi J, Sun W, and Kong X. 2024. The positive association between the atherogenic index of plasma and the risk of new-onset hypertension: a nationwide cohort study in China. *Clin Exp Hypertens* 46:2303999. 10.1080/10641963.2024.2303999

Zhang X, Ding Y, Shao Y, He J, Ma J, Guo H, Keerman M, Liu J, Si H, Guo S, and Ma R. 2021. Visceral Obesity-Related Indices in the Identification of Individuals with Metabolic Syndrome Among Different Ethnicities in Xinjiang, China. *Diabetes Metab Syndr Obes* 14:1609-1620. 10.2147/dmso.S306908
