# Supplementary material for: Diagnostic accuracy of the atherogenic index of plasma in metabolic syndrome: a diagnostic meta-analysis
Source: PeerJ. 2025 Oct 7;13:e20074. doi: 10.7717/peerj.20074 (PMC12513372; doi:10.7717/peerj.20074)
Supplement: Supplemental Information 3 [file peerj-13-20074-s003.docx]

**Table S1.** **The search strategy (Pubmed)**

| Search number | Query | Results |
| --- | --- | --- |
| 1 | Metabolic Syndrome[MeSH Terms]Program"[Title/Abstract] OR "Weight Lifting Exercise Program"[Title/Abstract] OR "Weight Lifting Strengthening Program"[Title/Abstract] OR "Yoga"[Title/Abstract] | 40216 |
| 2 | "Cardiometabolic Syndrome*"[Title/Abstract] OR "Dysmetabolic Syndrome X"[Title/Abstract] OR "insulin resistance syndrome"[Title/Abstract] OR "Insulin Resistance Syndrome X"[Title/Abstract] OR "Metabolic Cardiovascular Syndrome"[Title/Abstract] OR "Metabolic Syndrome"[Title/Abstract] OR "Metabolic Syndrome X"[Title/Abstract] OR "Metabolic Syndromes"[Title/Abstract] OR "Metabolic X Syndrome"[Title/Abstract] OR "MetSyn (metabolic syndrome)"[Title/Abstract] OR "Reaven syndrome"[Title/Abstract] OR "Reaven Syndrome X"[Title/Abstract] OR "Reaven's syndrome"[Title/Abstract] OR "Reaven's syndrome X"[Title/Abstract] OR "syndrome of insulin resistance"[Title/Abstract] | 2,733 |
| 3 | "atherogen index of plasma"[Title/Abstract] OR "atherogenic index"[Title/Abstract] OR "atherogenic index of plasma"[Title/Abstract] OR "atherogenicity index"[Title/Abstract] OR "atherogenicity index of plasma"[Title/Abstract] | 2406 |
| 4 | (#1 OR #2) AND #3 | 201 |
